# AIM2 Inflammasome Activation Leads to IL-1α and TGF-β Release From Exacerbated Chronic Obstructive Pulmonary Disease-Derived Peripheral Blood Mononuclear Cells

**DOI:** 10.3389/fphar.2019.00257

**Published:** 2019-03-15

**Authors:** Chiara Colarusso, Michela Terlizzi, Antonio Molino, Pasquale Imitazione, Pasquale Somma, Roberto Rega, Antonello Saccomanno, Rita P. Aquino, Aldo Pinto, Rosalinda Sorrentino

**Affiliations:** ^1^Department of Pharmacy, University of Salerno, Fisciano, Italy; ^2^ImmunePharma s.r.l., University of Salerno, Fisciano, Italy; ^3^PhD Program in Drug Discovery and Development, Department of Pharmacy, University of Salerno, Fisciano, Italy; ^4^Respiratory Division, Department of Respiratory Medicine, University of Naples Federico II, Naples, Italy; ^5^Department of Anatomy and Pathology, Ospedale dei Colli “Monaldi-CTO,” Naples, Italy

**Keywords:** chronic lung inflammation, COPD, inflammasome, IL-1-like cytokines, fibrosis

## Abstract

Chronic obstructive pulmonary disease (COPD) is now the fourth-leading cause of death worldwide and its prevalence is increasing. The progressive decline of lung function and airway remodelling are a consequence of chronic inflammatory responses. It was recently postulated the involvement of the inflammasome in COPD, although the underlying mechanism/s still need to be elucidated. Therefore, we isolated peripheral blood mononuclear cells (PBMCs) from exacerbated/unstable COPD patients. The stimulation of PBMCs with an AIM2 inflammasome activator, Poly dA:dT, led to IL-1α, but not IL-1β, release. The release of this cytokine was caspase-1- and caspase-4-dependent and correlated to higher levels of 8-OH-dG in COPD compared to non-smoker and smoker-derived PBMCs. Interestingly, AIM2-depedent IL-1α release was responsible for higher TGF-β levels, crucial mediator during pro-fibrotic processes associated to COPD progression. In conclusion, our data highlight the involvement of AIM2/caspase-1/caspase-4 in IL-1α-induced TGF-β release in unstable COPD-derived PBMCs, opening new therapeutic perspectives for unstable COPD patients.

## Introduction

Chronic Obstructive Pulmonary Disease (COPD) is a pulmonary disorder characterized by chronic lung and systemic inflammation, associated with progressive and irreversible decline of lung function, airway remodelling, and destruction of lung parenchyma and loss of alveolar attachments (Barnes, 2014). Inhalation of noxious indoor and outdoor particles or gas, especially cigarette smoke (CS) is the main risk factor for the development of this respiratory disorder (Caramori et al., 2016; De Falco et al., 2017a). Indeed, the site of deposition of inhaled irritants, among which urban particulate matter (PM), CS and particles from burning biomass fuels (Perez-Padilla et al., 2010; Barnes, 2014; Colarusso et al., 2017) is characterized by pro-inflammatory patterns, typical of COPD patients. In this scenario, we recently demonstrated that combustion-generated ultrafine particles (UFPs) induced COPD-derived peripheral blood mononuclear cells (PBMCs) to release IL-1-like cytokines, although in a non-canonical manner, via the involvement of caspase-4, rather than caspase-1 and -8 (De Falco et al., 2017a). Literature regarding the involvement of the inflammasome in COPD is still discordant (Colarusso et al., 2017). The inflammasome is a multiprotein complex that comprises the assembly of NLRs or HIN200 family receptors, able to bind the adaptor apoptosis-associated speck-like protein (ASC) that acts as a “zipper” and induces the auto-cleavage of procaspase-1, that in its active form facilitates the activation and then the release of IL-1-like cytokines (Terlizzi et al., 2014). Most of the studies on the involvement of inflammatory patterns in COPD focus on NLRP3 inflammasome. However, its involvement is controversial in that it depends on the exacerbation or stable status of the COPD patient (Colarusso et al., 2017). While some authors assume that NLRP3 is responsible for the release of high levels of IL-1β found in the lungs of COPD patients (Di Stefano et al., 2014; Kim et al., 2015), others demonstrated an indirect role of NLRP3 inflammasome in COPD (De Nardo et al., 2014; Di Stefano et al., 2014; Yang et al., 2015). Nevertheless, it was found that members of the IL-1 family cytokines, such as IL-1α and IL-1β are increased both in stable and in exacerbated COPD patients and might contribute to the detrimental chronic inflammation typical of this disease (Colarusso et al., 2017).

Therefore, the goal of our study was to understand the involvement of the inflammasome in COPD. We found that COPD-derived PBMCs produced higher levels of IL-1α than non-COPD in an AIM2-dependent manner. In particular, unstable COPD-derived PBMCs produced higher levels of TGF-β in an AIM2-caspase-1-caspase-4-dependent manner.

## Materials and Methods

### Human Samples

We used blood from exacerbated COPD and non-COPD patients recruited at the “Monaldi-Azienda Ospedaliera (AORN)-Ospedale dei Colli” Hospital in Naples, Italy, after their approval according to the Review Board of the hospital. Written informed consent was obtained from the participants of this study. The experimental protocol was performed in accordance with the guidelines and regulations provided by the Ethical Committee of the “Monaldi-Azienda Ospedaliera (AORN)-Ospedale dei Colli” (protocol n. 604/2017). Non-COPD patients were divided into 2 groups based on their smoking history: non-smokers and smokers; COPD patients were all smokers or former smokers. All the subjects were 50 ± 10 years of age and had no history of allergic diseases or chronic respiratory conditions. Supplementary Table S1 describes COPD patients according to their gender, smoking status, GOLD stage and pharmacological treatment. Blood was collected during exacerbation (when patients were hospitalized due to their low/altered pulmonary function) and after 3–5 days during stable (when patients were treated with endovenous corticosteroids and had stable respiratory functionality) status. Blood was used within 24 h in order to isolate mononuclear cells on which to evaluate the role of the inflammasome and its role in IL-1 signature.

### Isolation of Human PBMCs

Peripheral blood mononuclear cells (PBMCs) were isolated according to Ficoll’s protocol as already reported (De Falco et al., 2017b). Briefly, blood (5 ml) was mixed with cell medium (5 ml) supplemented with sole antibiotics and Ficoll medium (Life Sciences, Italy). PBMCs layer was collected and platelets were separated by centrifugation at 149 *g* for 10 min. PBMCs were then collected in cell medium, plated and treated for 1, 5 or 24 h accordingly. PBMCs were treated with the following substances: LPS 0.1 μg/ml, ATP 0.5 mM, Poly (dA:dT) (dA:dT) 1 μg/ml, Ac-Y-VAD-cmk (y-VAD) 1 μg/ml, Z-LEVD-FMK (z-LEVD) 10 μM, Pirfenidone (PIRF) (0.1 μg/ml) Nintedanib (10 nM), monoclonal antibody anti-IL-1α (α-IL-1α) (1 ng/ml). Concentrations of the above treatments were chosen according to published data (Sorrentino et al., 2015; Terlizzi et al., 2016, 2018b; De Falco et al., 2017a). PBMCs were treated for 5 or 24 h according to the experimental protocol. Cell viability was evaluated by means of MTT assay. No changes in the optical density (OD) at 550 nm were observed (CTR: 0.568 ± 0.03; dA:dT: 0.610 ± 0.04 at 5 h; CTR: 0.591 ± 0.03; dA:dT: 0.600 ± 0.033 at 24 h).

### Cytokine Measurements

IL-1α and TGF-β were measured in cell-free supernatants obtained from the PBMCs culture, respectively, after 5 and 24 h of treatment, using commercially available enzyme-linked immunosorbent assay kits (ELISAs) (eBioscience, CA, United States; R&D Systems, United States).

The level of 8-OH-dG was measured following manufacturer’s instructions (Elabscience, Houston, TX United States) after 1 h of treatment.

### Flow Cytometry Analysis

AIM2 expression was performed by flow cytometry (BD FacsCalibur, Milan, Italy) by staining untreated PBMCs with the following antibodies: AIM2-FITC and CD14-PE (eBioscience, San Diego, CA, United States). PBMCs were stained for the extracellular CD14 and then fixed and permeabilized by means of BD Cytofix/Cytoperm solutions before adding anti-AIM2 antibody.

### Statistical Analysis

Data are reported as the median ± interquartile range. Each experiment was performed in duplicate. Statistical differences were assessed with ONE-Way ANOVA followed by multiple comparisons Bonferroni’s post-test or Mann-Whitney U test as non-parametric Student’s *t* test as appropriate. *p* values less than 0.05 were considered as significant.

## Results

### The Activation of AIM2, but Not NLRP3, Inflammasome Drives to IL-1α Release in Exacerbated/Unstable COPD-Derived PBMCs

**FIGURE 1:**
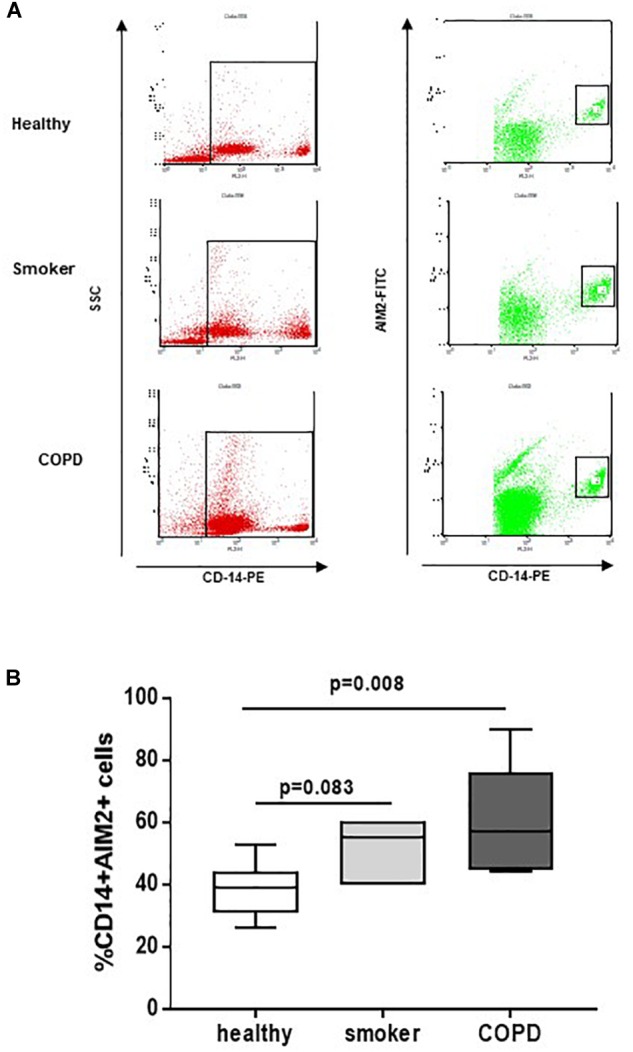
COPD-derived CD14^+^ PBMCs express higher levels of AIM2. Isolated PBMCs from healthy non-smokers, smokers and COPD patients were analyzed by flow cytometry for CD14 and AIM2 expression, based on SSC-CD14+ gate. **(A)** Representative flow cytometry data expressed in the graph below **(B)**. Data are represented as median ± interquartile range (*n* = 7). Statistically significant differences were determined by one-way ANOVA followed by Bonferroni’s multiple comparison post-test.

In our previous study, we found that NLRP3 expression is statistically increased in PBMCs of COPD patients (De Falco et al., 2017a). Here, we found that, similarly, AIM2 expression in CD14+ PBMCs (Figure 1A,B) was statistically increased in COPD patients compared to smokers and healthy subjects (Figure 1B).

**FIGURE 2:**
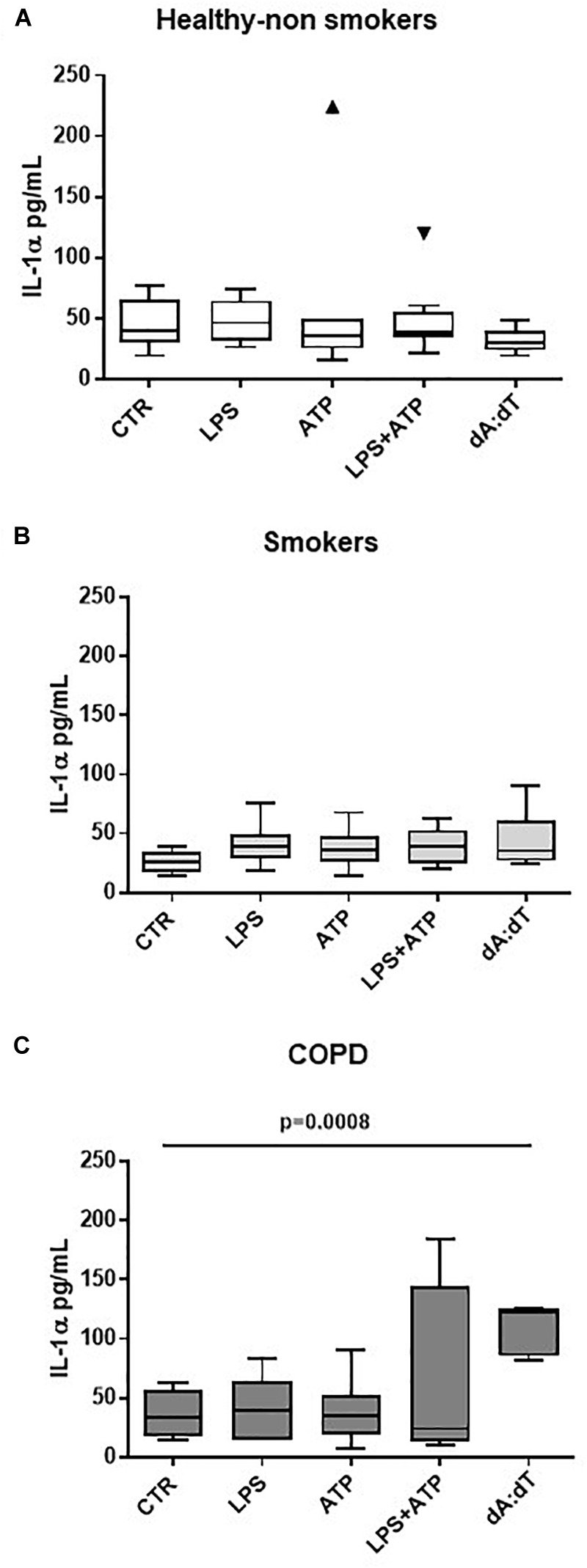
IL-1α release from unstable COPD-derived PBMCs is induced after AIM2 inflammasome activation. The addition of Poly dA:dT (dA:dT, 1 μg/ml), AIM2 inducer, to PBMCs obtained from healthy non-smokers **(A)**, smokers **(B)** and unstable COPD **(C)** patients, significantly increased IL-1α release from COPD-derived PBMCs compared to smokers and non-smokers. Data are represented as median ± interquartile range (*n* = 10 independent subjects). Statistically significant differences were determined by one-way ANOVA followed by Bonferroni’s multiple comparison post-test.

Therefore, in order to understand the involvement of both inflammasomes, we triggered NLRP3 with LPS ± ATP, and AIM2 with Poly dA:dT (dA:dT). We found that healthy- (Figure 2A) and smoker-derived PBMCs (Figure 2B) were not able to release IL-1α neither after NLRP3 nor after AIM2 triggering. In sharp contrast, the sole stimulation of AIM2 via the addition of dA:dT (1 μg/ml) significantly increased the release of IL-1α from unstable COPD-derived PBMCs (Figure 2C).

Importantly, although its higher expression (De Falco et al., 2017a), we did not observe statistical differences in IL-1α levels after the activation of NLRP3 via the addition of LPS (0.1 μg/ml) ± ATP (0.5 mM), according to the two-signal inflammasome activation mode (Terlizzi et al., 2014; Colarusso et al., 2017). Similarly, we did not observe statistical differences in IL-18 and IL-1β release when LPS+ATP or dA:dT were added (Supplementary Figures S1A,B), implying that NLRP3 was not functional, as already published (Terlizzi et al., 2018b).

**FIGURE 3:**
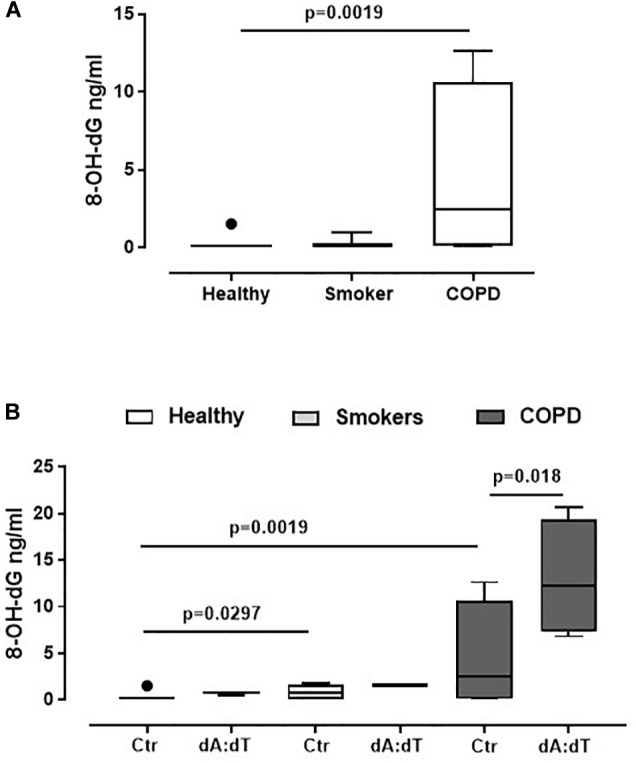
AIM2 stimulation in unstable COPD-derived PBMCs leads to higher levels of 8-OH-dG. **(A)** Levels of 8-OH-dG were analyzed by means of ELISA in PBMCs from healthy non-smokers, smokers and COPD subjects. **(B)** Stimulation of AIM2 via Poly dA:dT (dA:dT, 1 μg/ml) statistically increased the levels of 8-OH-dG. Data are represented as median ± interquartile range (*n* = 5). Statistically significant differences were determined by **(A)** one-way ANOVA followed by Bonferroni’s multiple comparison post-test or **(B)** Mann Whitney U Test.

Because AIM2 recognizes cytosolic dsDNA (Jin et al., 2012; Terlizzi et al., 2014), in order to understand the relationship between oxidative stress, crucial in the lung of COPD patients (Faner et al., 2016; Colarusso et al., 2017), and AIM2, we analyzed the levels of 8-OH-dG, which is a marker for oxidative stress to DNA (Halwani et al., 2011; De Falco et al., 2017a). We found that COPD-derived PBMCs had higher basal levels of 8-OH-dG (Figure 3A) than healthy non-smokers and smokers (Figure 3A). In addition, the stimulation of AIM2 further increased the levels of 8-OH-dG in COPD-derived PBMCs (Figure 3B). Instead, the stimulation of both healthy- and smoker-derived PBMCs did not reach statistical significance after Poly dA:dT addition (Figure 3B), further highlighting the relevance of AIM2 in COPD-associated inflammation.

### AIM2 Inflammasome Activation Induces IL-1α Release in a Caspase-1-/Caspase-4-Dependent Manner

**FIGURE 4:**
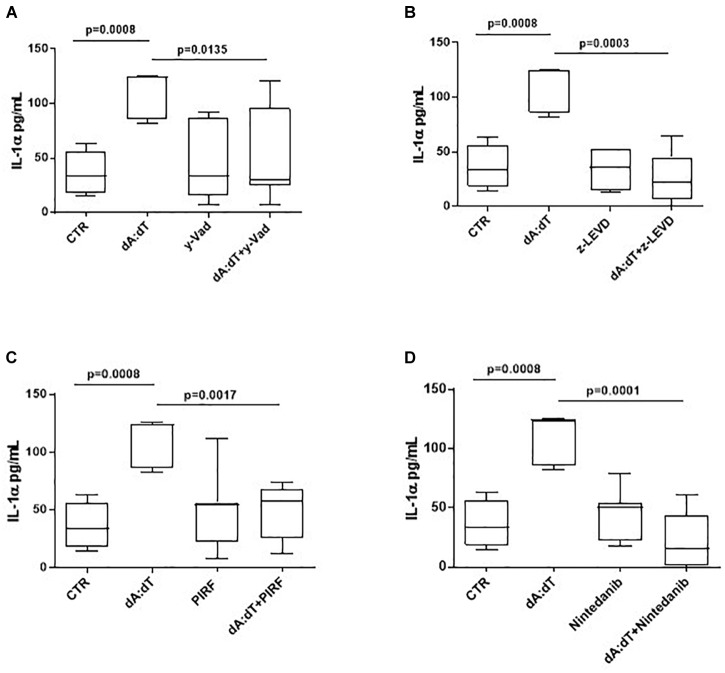
The release of IL-1α after AIM2 stimulation was caspase-1 and caspase-4 dependent in unstable COPD-derived PBMCs. The addition of y-VAD, caspase-1 inhibitor **(A)**, z-LEVD, caspase-4 inhibitor **(B)**, Pirfenidone (PIRF) **(C)** and Nintedanib **(D)** significantly reduced IL-1α release after AIM2 activation by means of Poly dA:dT (dA:dT). Data are represented as median ± interquartile range (*n* = 10). Statistically significant differences were determined by one-way ANOVA followed by Bonferroni’s multiple comparison post-test.

In order to understand the molecular mechanisms underlying IL-1α release after AIM2 stimulation, we went on to evaluate whether caspase-1, associated to the canonical inflammasome, as well as caspase-4, associated to the non-canonical inflammasome, were involved. Therefore, we used pharmacological inhibitors of caspase-1 and caspase-4. In particular, we observed that when PBMCs were co-treated with dA:dT and Ac-Y-VAD-cmk (y-VAD, 1 μg/ml), a well-known caspase-1 pharmacological inhibitor (Sorrentino et al., 2015; Terlizzi et al., 2016, 2018b; De Falco et al., 2017a,b), the release of IL-1α was statistically decreased (*p* = 0.0135) (Figure 4A). Similarly, the pharmacological inhibition of caspase-4 by means of Z-LEVD-FMK (z-LEVD, 10 μM) (Eric, 2011) robustly reduced the release of IL-1α after dA:dT treatment (Figure 4B).

Based on the concept that COPD is characterized by lung fibrosis associated with remodeling of small airways (Barnes, 2014) and that IL-1-like cytokines are involved in this process (Borthwick, 2016), and because we already found that the activation of the AIM2 inflammasome is involved in a pro-fibrotic process (Terlizzi et al., 2018b), we evaluated whether AIM2-dependent IL-1α release in COPD-derived PBMCs were impaired after treatment with two different anti-fibrotic drugs (Halwani et al., 2011). The addition of both Pirfenidone (PIRF, 0.1 μg/ml) (Figure 4C), an inhibitor of TGF-β release (Terlizzi et al., 2018b), and Nintedanib (10 nM) (Figure 4D), a small molecule able to inhibit tyrosine kinase cascade (Terlizzi et al., 2018b), significantly decreased IL-1α levels from COPD-derived PBMCs treated with dA:dT.

Taken together, these results indicate that AIM2 inflammasome pathway is activated in COPD-derived PBMCs and is responsible for IL-1α release in a caspase-1- and caspase-4 dependent manner, most likely via the involvement of tyrosine kinase that are involved in pro-fibrotic patterns.

### Stimulation of AIM2 Inflammasome Triggers TGF-β Release From COPD-Derived PBMCs in a Caspase-1- and Caspase-4-Dependent Manner

Progressive small-airway fibrosis is an important mechanism in the progression of COPD and is caused by fibrogenetic mediators-induced fibroblasts activation (Liebhart and Dobek, 2009; Barnes, 2014; Borthwick, 2016). Thus, we focused our attention on the role of AIM2/IL-1α axis on the release of TGF-β, which is fundamental for both lung inflammation and remodelling (Sheppard, 2006; Halwani et al., 2011).

**FIGURE 5:**
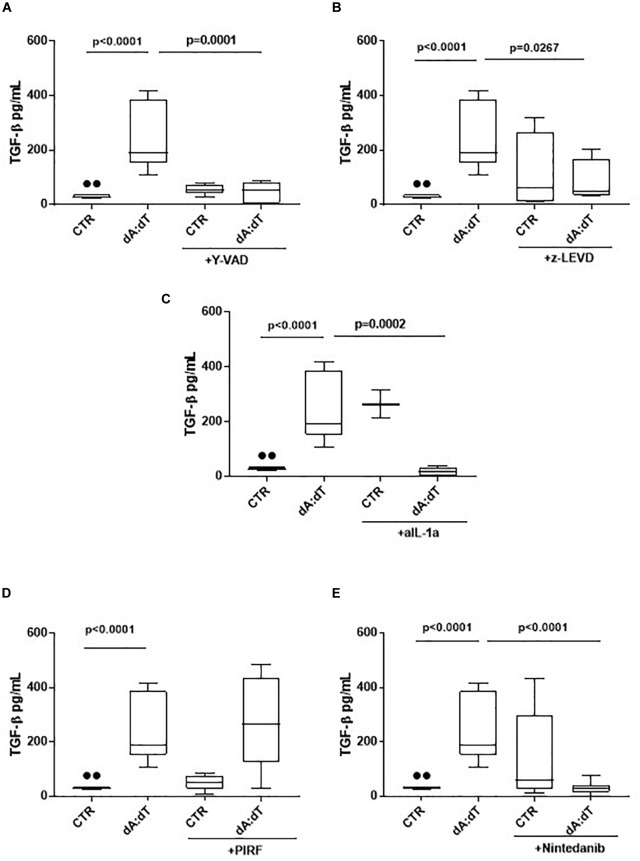
The release of TGF-β after AIM2 stimulation was dependent on caspase-1, caspase-4 and IL-1α in unstable COPD-derived PBMCs. The addition of y-VAD, caspase-1 inhibitor **(A)**, z-LEVD, caspase-4 inhibitor **(B)**, monoclonal antibody against IL-1α **(C)**, Nintedanib **(E)**, but not Pirfenidone (PIRF) **(D)**, significantly reduced TGF-β release after AIM2 activation at 24 h post-treatment. Data are represented as median ± interquartile range (*n* = 10). Statistically significant differences were determined by one-way ANOVA followed by Bonferroni’s multiple comparison post-test.

The activation of AIM2 by means of dA:dT robustly increased the levels of TGF-β (Figure 5A). In order to understand whether the previous observed caspase-1/caspase-4/IL-1α axis was involved, we analyzed the levels of TGF-β at 24 h post the addition of y-VAD, a caspase-1 inhibitor, or z-LEVD, a caspase-4 inhibitor. Again, we observed that the inhibition of both caspase-1 (Figure 5A) and caspase-4 (Figure 5B) significantly reduced the levels of TGF-β from COPD-derived PBMCs. Because these two enzymes are likely to be upstream IL-1α release (Gross et al., 2012; Casson et al., 2013; Terlizzi et al., 2014), we used a neutralizing antibody for IL-1α as previously reported (Terlizzi et al., 2018b). The antibody-mediated neutralization of IL-1α significantly reduced the release of TGF-β (Figure 5C), implying that the activation of caspase-1 and caspase-4 following AIM2 triggering, induced IL-1α to promote TGF-β release. The isotype control IgG did not alter the levels of basal TGF-β (27.5 ± 3.67 pg/ml data not shown). To prove of this interpretation, we used Pirfenidone, which is reported to inhibit TGF-β release in fibrotic processes down-regulating its transcription (Knüppel et al., 2017).

Interestingly, the co-treatment with Pirfenidone in the presence of dA:dT did not alter TGF-β levels (Figure 5D), most likely due to the prevalence of IL-1α-dependent signaling. Indeed, the addition of Nintedanib, tyrosine kinase inhibitor, significantly reduced TGF-β levels after AIM2 stimulation (Figure 5E), implying the role of these enzymes in the induction of TGFβ levels. Nevertheless, it has to be pointed out that although Pirfenidone was able to inhibit IL-1α release after AIM2 activation, the release of TGFβ was not blocked by this drug at 24 h, implying that most likely other signaling pathways could lead to TGFβ release after AIM2 activation. Therefore, further investigations will be needed.

Based on these data we propose another molecular mechanism underlying the release of TGF-β. The activation of AIM2 inflammasome leads to caspase-1 and caspase-4 activation responsible for IL-1α release, which leads to TGF-β release from COPD-derived PBMCs.

## Discussion

Recent evidence highlight the involvement of the multiprotein complex inflammasome in COPD onset and progression (Colarusso et al., 2017).

In this study we found a novel molecular mechanism that leads to IL-1α release from COPD-derived PBMCs, responsible for the induction and release of TGF-β, an immune suppressive and pro-fibrotic cytokine (André et al., 2015; Nigdelioglu et al., 2016; Terlizzi et al., 2018b). In particular, we found that AIM2 inflammasome is activated in exacerbated/unstable COPD-derived PBMCs and leads to IL-1α and TGF-β release in a caspase-1- and caspase-4-dependent manner.

Several clinical studies have demonstrated that IL-1-like cytokines levels are elevated in sputum and in broncho-alveolar lavage (BAL), and that these levels augment during exacerbation (Borthwick, 2016; Colarusso et al., 2017). In support, Franklin et al. (2014) reported that NRLP1, AIM2 and NLRP3 inflammasome activation could mediate the accumulation of ASC specks, relevant for COPD establishment. Indeed, BAL of CS-induced COPD mouse model and the lungs, sputum and PBMCs from COPD patients were rich of ASC specks that recruited caspase-1 to boost IL-1β activation (Franklin et al., 2014). Similarly, we found that IL-1α, but not IL-1β, is significantly released from unstable COPD-derived PBMCs after AIM2, but not NLRP3, activation, which could be an autocrine activation due to the higher levels of oxidative stress-derived DNA motifs in COPD. Basal levels of 8-OH-dG, marker of DNA oxidative stress (Valavanidis et al., 2009; Halwani et al., 2011; De Falco et al., 2017a), were higher in COPD-derived PBMCs than healthy non-smokers and smokers, implying that DNA damage during the pro-inflammatory patterns, could boost AIM2 activation in these patients. Moreover, stimulation of AIM2 further increased the levels of 8-OH-dG in COPD-derived PBMCs compared to healthy and smokers. These data are in accordance with what already published (De Falco et al., 2017a) in that healthy and smokers have higher levels of OGG1, a repairing DNA enzyme, compared to COPD-derived PBMCs, who have reduced levels of the enzyme to counter the oxidative stress damage to DNA due to higher levels of ROS. In this scenario, AIM2 activation is involved in COPD exacerbation via the release of IL-1α, responsible for the induction of TGF-β, immune suppressive and pro-fibrotic cytokine. Similarly, in our previous work, we found that IL-1α was responsible for TGF-β release from idiopathic pulmonary fibrosis (IPF)-derived PBMCs in a caspase-4-, but not caspase-1-, dependent manner (Terlizzi et al., 2018b). In this study, instead, we found that AIM2-induced IL-1α release was inhibited by both y-Vad, caspase-1 inhibitor, and z-LEVD, caspase-4 inhibitor. Because caspase-4 has been described to be upstream caspase-1 activation (Sollberger et al., 2012), we speculate that AIM2 activation leads first to caspase-4 and then to caspase-1 activation, although the exact mechanism still needs to be elucidated. The limitation to pursue this goal is the nature of the primary cells which are difficult to genetically manipulate, and to the still unknown endogenous ligand that can lead to caspase-4 activation. In this regard, it was reported that LPS could serve as intracellular inducer of caspase-4 (Shi et al., 2014). Nevertheless, in our study, we did not find an increase of IL-1α when LPS was administered, implying that it first does not induce IL-1α release via caspase-4, and then suggesting that NLRP3, which was described to be activated by the murine analog caspase-11 (Terlizzi et al., 2014), is not induced by caspase-4 in these cells. In contrast, AIM2-induced IL-1α was inhibited after the addition of z-LEVD, a caspase-4 inhibitor. Therefore, we could speculate that the activation of AIM2 leads to caspase-4 and then caspase-1 activation in that to induce IL-1α release. In support, it was recently demonstrated that IL-1α can be processed by caspase-11 in mice (homolog of caspase-4 in humans) via a non-canonical inflammasome (Casson et al., 2013). Moreover, we found that the inhibition of caspase-4 by means of z-LEVD (Figure 4B) completely reduced the levels of IL-1α compared to y-VAD, caspase-1 inhibitor (Figure 4A), implying that caspase-4 is upstream caspase-1 for the induction of IL-1α when AIM2 is activated. Here, we found that caspase-4 mRNA levels were higher in COPD-derived PBMCs than smokers and non-smokers (data not shown; COPD: median = 14.12 ratio caspase-4/β-actin mRNA; Smokers: median = 4.971 ratio caspase-4/β-actin mRNA, Healthy non-smoker: median = 0.9059 ratio caspase-4/β-actin mRNA). In another study, we found that the levels of the circulating caspase-4 were detectable in the plasma of COPD patients (Terlizzi et al., 2018a), further confirming the involvement of this enzyme in COPD. In contrast, Eltom et al. (2014) found that caspase-11, the murine analog of caspase-4, was not involved in lung inflammation in a mouse model of CS-driven acute COPD. However, this discrepancy probably stands on the difference between mice and humans.

To date, this is the first study, to our knowledge, that focuses on the activation of AIM2 in COPD-derived PBMCs. Most of the literature has been focused on NLRP3, which role is still controversial, probably due to the status of COPD patients (stable vs. exacerbated). In this study, we did not find NLRP3 involvement. Similarly, in our previous report we found that inflammasome activation by urban particulate-like matter was not NLRP3-dependent (De Falco et al., 2017a). However, it is possible to speculate that, because 8-OH-dG sequesters NLRP3 (Eltom et al., 2014), it is likely that this receptor is not responsive in COPD-derived PBMCs, which have higher basal levels of 8-OH-dG, marker of DNA damage associated to oxidative stress, one of the main feature of chronic inflammation in COPD (Colarusso et al., 2017).

Several studies have shown that IL-1α is important in lung inflammation and fibrosis (Borthwick, 2016). Fibrotic processes play a pivotal role in COPD causing significant lung dysfunction by remodeling small airways and contributing to airflow limitation (Barnes, 2014). During lung damage, epithelial cells release high amount of pro-inflammatory mediators (Issa et al., 2008), among which, active IL-1α acts as primary fibroblast stimulatory factor, directly stimulating collagen synthesis and proliferation (Borthwick, 2016). Here we found that among two anti-fibrotic drugs (Pirfenidone and Nintedanib) the sole Nintedanib abrogated AIM2/ IL-1α-dependent TGF-β release from PBMCs of COPD patients. To note, very relevant was that IL-1α was responsible for TGF-β release at 24 h, effect that was again dependent on caspase-1 and caspase-4, as well as on tyrosine kinase cascade. Progressive small airway fibrosis (Vignola et al., 1996; Thomas et al., 2016) is a feature of COPD and it is associated to chronic inflammation (Barnes, 2014). During fibrotic events a critical role is played by fibrotic mediators secreted by macrophages and epithelial cells. The main pro-fibrotic mediator is TGF-β, critical for the expression of collagen genes and mesenchymal cell-related markers, such as α-smooth muscle actin (α-SMA) and vimentin (Liebhart and Dobek, 2009; Borthwick, 2016).

Collectively, these data suggest that the activation of AIM2/caspase-4/caspase-1 axis leads to IL-1α release which is responsible for TGF-β release from PBMCs. To note, TGF-β release was not altered by Pirfenidone, which mechanism of action is to inhibit TGF-β-induced fibroblast proliferation (Conte et al., 2014). These latter data further support the specificity of AIM2-induced IL-1α in unstable COPD-derived PBMCs, another source of TGF-β. To note, stable COPD-derived PBMCs were not responsive to Poly dA:dT in that they showed no significant release of IL-1α (ctr: 23.61 ± 0.569 vs. dA:dT: 26.4 ± 0.872) compared to unstable COPD-derived PBMCs (ctr: 37.39 ± 5.486 vs. dA:dT: 234.9 ± 30.75; Figure 2C).

## Conclusion

In conclusion our study highlights a novel molecular mechanism by which canonical, caspase-1-, and non-canonical, caspase-4-, dependent AIM2 inflammasome leads to IL-1α that in turn induces TGF-β release, responsible for pro-fibrotic processes. This study paves the way for novel therapeutic perspectives/options, focusing on the biology of AIM2 inflammasome as involved during the exacerbation status of COPD patients.

## Data Availability

All datasets generated for this study are included in the manuscript and/or the Supplementary Files.

## Author Contributions

CC and MT performed the experiments. AM, PI, RR, and PS performed the diagnostic analyses. AS performed the statistical analyses. RA and AP edited the manuscript. RS designed the experiments, analyzed and interpreted the data, and wrote the manuscript. RR helped in recruiting human samples from COPD patients.

## Conflict of Interest Statement

The authors declare that the research was conducted in the absence of any commercial or financial relationships that could be construed as a potential conflict of interest.

## Supplementary Material

Figure S1 Release of IL-18 **(A)** and IL-1β **(B)** after LPS ± ATP or Poly dA:dT addition to unstable COPD-derived PBMCs. Data are represented as median ± interquartile range (*n* = 5).

Table S1 COPD Patients’ information.
