# Supplementary figures and images for: AIM2 Inflammasome Activation Leads to IL-1α and TGF-β Release From Exacerbated Chronic Obstructive Pulmonary Disease-Derived Peripheral Blood Mononuclear Cells
Source: Front Pharmacol. 2019 Mar 15;10:257. doi: 10.3389/fphar.2019.00257 (PMC6428726; doi:10.3389/fphar.2019.00257)

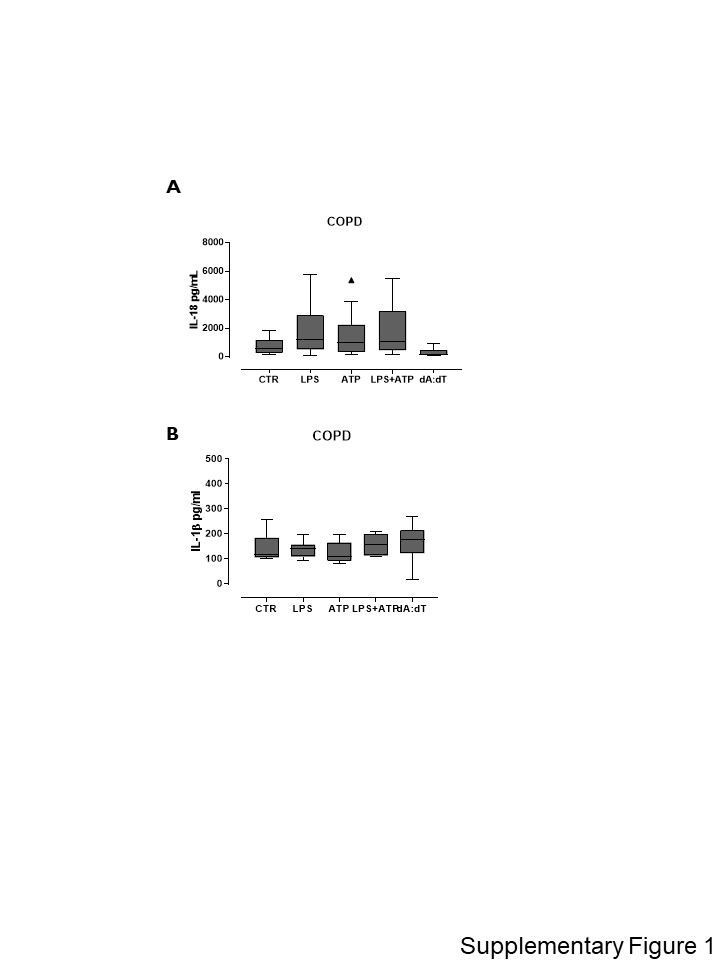

Supplement: Figure S1 — Release of IL-18 (A) and IL-1β (B) after LPS ± ATP or Poly dA:dT addition to unstable COPD-derived PBMCs. Data are represented as median ± interquartile range (n = 5). [file Image_1.JPEG]

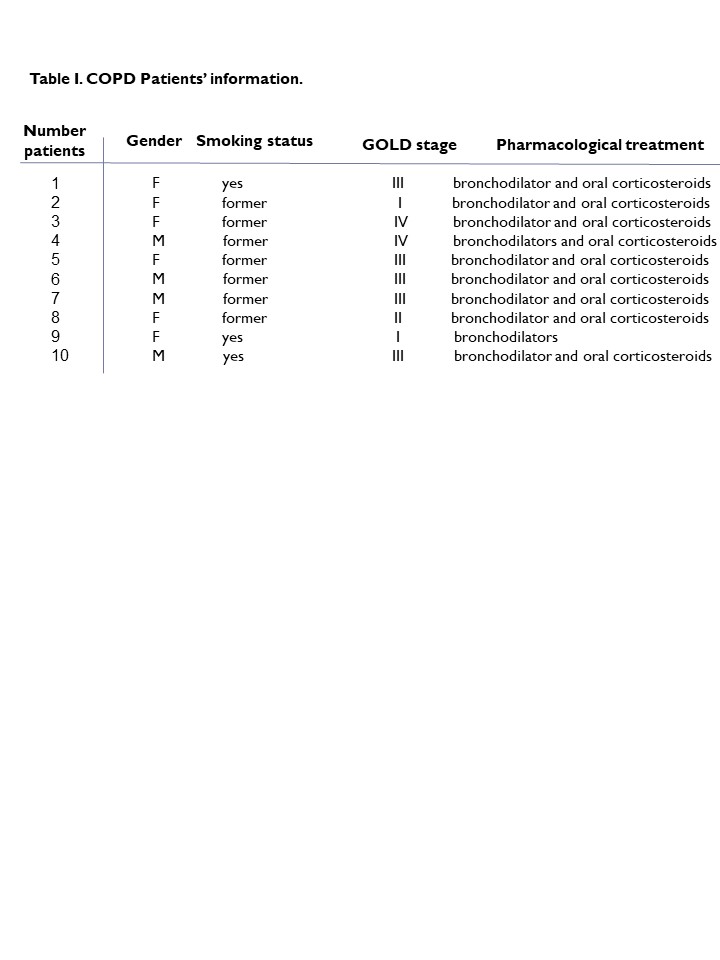

Supplement: Table S1 — COPD Patients’ information. [file Image_2.jpg]
